# Noninvasive Contrast-Free 3D Evaluation of Tumor Angiogenesis with Ultrasensitive Ultrasound Microvessel Imaging

**DOI:** 10.1038/s41598-019-41373-0

**Published:** 2019-03-20

**Authors:** Chengwu Huang, Matthew R. Lowerison, Fabrice Lucien, Ping Gong, Diping Wang, Pengfei Song, Shigao Chen

**Affiliations:** 1 Department of Radiology, Mayo Clinic College of Medicine and Science, Mayo Clinic, Rochester, MN USA; 2 Department of Urology, Mayo Clinic College of Medicine and Science, Mayo Clinic, Rochester, MN USA; 3 Department of Laboratory Medicine and Pathology, Mayo Clinic College of Medicine and Science, Mayo Clinic, Rochester, MN USA

## Abstract

Ultrasound microvessel imaging (UMI), when applied with ultrafast planewave acquisitions, has demonstrated superior blood signal sensitivity in comparison to conventional Doppler imaging. Here we propose a high spatial resolution and ultra-sensitive UMI that is based on conventional line-by-line high-frequency ultrasound imagers and singular value decomposition (SVD) clutter filtering for the visualization and quantification of tumor microvasculature and perfusion. The technique was applied to a chicken embryo tumor model of renal cell carcinoma that was treated with two FDA-approved anti-angiogenic agents at clinically relevant dosages. We demonstrate the feasibility of 3D evaluation with UMI to achieve highly sensitive detection of microvasculature using conventional line-by-line ultrasound imaging on a preclinical and commercially available high-frequency ultrasound device without software or hardware modifications. Quantitative parameters (vascularization index and fractional moving blood volume) derived from UMI images provide significantly improved evaluation of anti-angiogenic therapy response as compared with conventional power Doppler imaging, using histological analysis and immunohistochemistry as the reference standard. This proof-of-concept study demonstrates that high-frequency UMI is a low-cost, contrast-agent-free, easily applicable, accessible, and quantitative imaging tool for tumor characterization, which may be very useful for preclinical evaluation and longitudinal monitoring of anti-cancer treatment.

## Introduction

The accurate detection and quantification of small slow-flow blood vessels provides a biomarker of vascular perfusion, which has been shown to be a critical read-out in the diagnosis and monitoring of many pathological disease states^1^. This is particularly true in the study of cancer, which is typified by the development of aberrant vasculature and perfusion defects^2^. Many cancer subtypes, such as renal cell carcinoma, are clinically treated with FDA-approved anti-angiogenic tyrosine kinase inhibitors (TKIs) in the front-line setting. Classically, these therapies are thought to directly antagonize the development of a supporting vascular bed^3,4^, with vascular normalization^5^ remaining a controversial hypothesis. However, conventional treatment response criteria, such as RECIST guidelines (Response Evaluation Criteria In Solid Tumours - version 1.1^6^), are insensitive to anti-angiogenic therapy effects, as reductions in tumor size can take several months to manifest^7^, and do not consider cytostatic agent activity that does not directly influence anatomical size^8^. There is considerable heterogeneity in individual RECIST responses to anti-angiogenic therapies, which are heavily influenced by tumor type and angiogenic features^9^, leading to great interest in the pursuit of complementary biomarkers. Furthermore, intratumoral hypoxia—a consequence of poor or aberrant vascular perfusion—has been linked to clinical resistance to more conventional cytotoxic therapies, such as chemotherapy, radiotherapy, and immunotherapy^10–13^. Therefore, quantitative evaluation of tumor microvasculature has important applications in cancer treatment response monitoring.

The need for microvasculature quantification in animal cancer models has motivated the development of commercially available and dedicated preclinical high-frequency ultrasound systems, which provide high-resolution anatomical and vascular (Doppler) images at a low relative cost and without ionizing radiation^14^. However, conventional Doppler imaging with these systems often has a low sensitivity to slow flow vessels. These limitations are partly due to the short Doppler ensemble length^15^ and the inability of traditional clutter filtering, which is based on high-pass temporal filtering^16^, to distinguish between microvasculature and tissue clutter. This limitation was addressed by the emergence of ultrafast ultrasound microvessel imaging (UMI), which combines the benefits of high frame-rate ultrasound plane wave imaging and Eigen-based tissue clutter filters^17,18^. High frame-rate plane-wave imaging enables the collection of a large number of Doppler ensembles in a short period of time, which can substantially increase Doppler sensitivity to slow flow signal from small vessels^15^. The rich spatiotemporal information offered by ultrafast ultrasound imaging also permits more robust tissue clutter rejection through advanced Eigen-based clutter filters—such as singular value decomposition (SVD)—that capitalize on the underlying differences in spatiotemporal characteristics between tissue, blood, and electronic noise^17^. Demené *et al*. have performed high sensitivity functional ultrasound imaging on murine brains through the combination of SVD clutter filtering with plane wave imaging^18^, and we have previously presented a series of improved SVD-based clutter filtering techniques with improved sensitivity and robustness^19^, computational speed^20^, and noise suppression performance^21^. However, most plane wave imaging systems for UMI are designed for human use and operate in the 3–15 MHz center frequency range, resulting in an imaging resolution that is not fine enough to visualize tumor microvascular structures or monitor the minor vascular effects induced by vessel targeting anti-cancer therapies in small animal models. These systems are limited by their analog-to-digital conversion sampling rate which prohibits plane wave imaging at very high center frequencies (>30 MHz). Some UMI systems (e.g.: the Verasonics Vantage 256) can overcome the upper bandwidth determined by their analogue-to-digital sampling rate via “interleaved acquisition sampling”, but these systems generally cannot support frequencies greater than 30 MHz, and such sampling techniques greatly increase the complexity of UMI imaging. It is therefore not trivial to perform ultrafast plane wave imaging in the high frequency range using existing clinical ultrafast imagers. Furthermore, ultrafast plane wave imaging is currently not available on commercial preclinical high-frequency ultrasound scanners—a situation that may hinder the translation of UMI into preclinical research on animal models. Therefore, the large installed base of small animal scanners that use conventional beam-forming techniques presents an unmet need for improved microvessel imaging using existing line-by-line scanning architecture of these scanners.

In this paper we present an ultrasensitive and high resolution UMI method using SVD-based clutter filtering on high-frequency (50 MHz) ultrasound IQ signal from mechanically swept 3D imaging volumes acquired using a commercially available pre-clinical ultrasound system (FUJIFILM VisualSonics Vevo^®^ 3100) without hardware or software modifications. Ultrasound IQ data is readily accessible with the Vevo^®^ 3100 system provided that the user has the commercially available ‘RF capture module’ license. With access to the RF data of the VisualSonics system, one can use the proposed algorithms to conveniently perform ultrasensitive UMI with existing equipment. In principle, the proposed algorithms should be applicable to any ultrasound system if the user has access to pre-envelope-compressed ultrasound data (either RF or IQ data).

The proposed UMI was applied to evaluate the microvasculature and perfusion of a chicken embryo chorioallantoic membrane (CAM) tumor model of renal cell carcinoma that was generated with Renca cancer cells (ATCC^®^ CRL-2947^TM^, *Mus musculus* renal adenocarcinoma). The Renca cell line, established from a spontaneous murine renal adenocarcinoma, has been successfully used as murine subcutaneous and orthotopic (renal capsule) tumor models, and as a pulmonary metastatic tumor model when seeded via tail vein injection^22^. We demonstrated that the chicken embryo CAM Renca tumor model permitted fast evaluation of intratumoral specimen responses to various targeted therapies, making it the ideal preclinical model for optimizing targeted therapy. We challenged the vascular development of this tumor model with the administration of two FDA-approved anti-angiogenic agents, sunitinib and pazopanib, at clinically relevant dosages. We demonstrated that high frequency UMI greatly improved the detection of microvasculature over conventional Doppler imaging and validated the results with gold-standard histological analysis and immunohistochemistry. These high resolution and ultrasensitive UMI images were produced using a preclinical device without the need for injection of microbubble contrast agents, a distinct advantage in the chicken embryo tumor model in which contrast injections for ultrasound localization microscopy^23^ are technically challenging. This easily applicable technique is a quantitative, reproducible, and accessible imaging tool for tumor vasculature and perfusion characterization, permitting longitudinal monitoring and prediction of treatment responses.

## Results

### The Renca cell line had a high engraftment efficiency on the CAM

**Figure 1:**
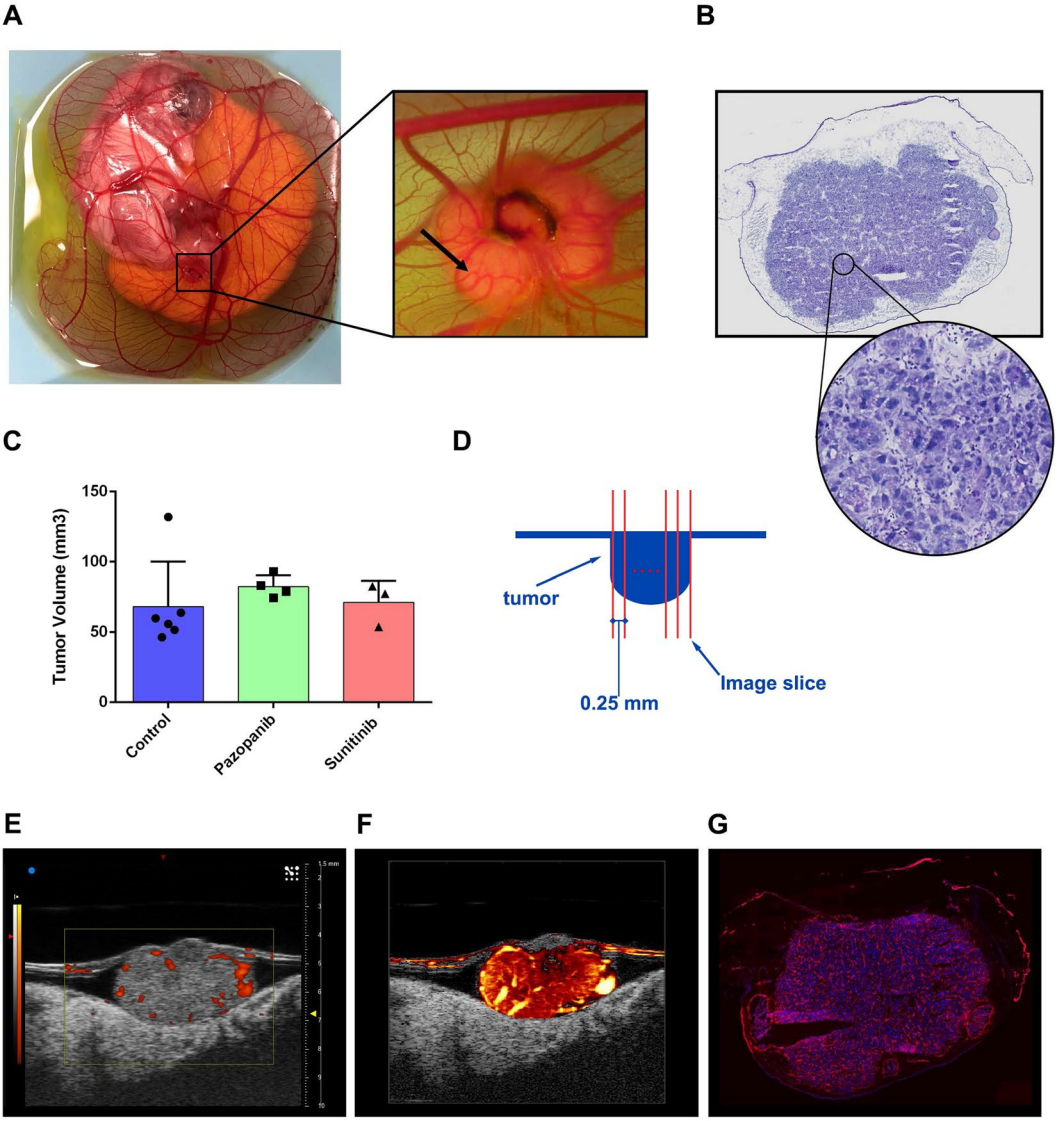
Functional imaging of CAM engrafted cell-line tumors. (**A**) Overhead view of an *ex ovo* chicken embryo model. Cancer cell lines engrafted into the CAM resulted in highly vascularized, solid, and spheroidal tumor masses. (**B**) H&E section taken from representative control tumor (DMSO treated). High magnification section reveals viable tumor nuclei with a high degree of adjacent vasculature. (**C**) Endpoint tumor volumes for each treatment group. Differences in volume were not found to be significant. Data are mean ± s.d. (**D**) Schematic representation of tumor imaging protocol. Tumors were imaged throughout their volume with a step size of 0.25 mm. (**E**) Representative image of control (DMSO) treated Renca tumor obtained with conventional power Doppler of VisualSonics Vevo^®^ 3100. (**F**) SVD-based clutter filtering of ultrasound echoes from the tumor in (**E**) resulted in improved vascular imaging (Supplementary Video S1). (**G**) Fluorescent histology section of the example tumor, which confirms high intratumoral vascularization (red signal).

Renca cells readily formed spheroidal tumors when inoculated into the CAM of chicken embryos, as demonstrated in Fig. 1A, with the majority of the tumor mass growing below the surface of the membrane. All of the engrafted embryos demonstrated visible tumor masses for the first day of therapy occurring on embryonic development day 11 (EDD-11), indicating high engraftment efficiency of this cell line on the CAM. The high engraftment efficiency was confirmed using H&E histology, which revealed viable cancer cell tissue throughout the tumor mass (Fig. 1B). However, the attrition rate of chick embryos was substantial, especially for the sunitinib treated cohort in which only three out of the original eight (3/8) engrafted embryos survived to the imaging end-point. The dimethyl sulfoxide (DMSO 1%) vehicle control group and pazopanib treated cohorts had 6/8 and 5/8 embryos survive until ultrasound imaging on EDD-17, respectively. The endpoint volumes for each of the tumor cohorts were 68.28 ± 28.97 mm^3^ for control, 83.10 ± 7.11 mm^3^ for pazopanib, and 71.36 ± 10.76 mm^3^ for sunitinib (Fig. 1C). This difference in tumor volumes was not found to be significant using a one-way ANOVA.

### CAM implanted Renca tumors were hyper-vascularized

Visually, CAM Renca tumors displayed several hallmarks of a large amount of pro-angiogenic signaling and neo-angiogenesis. These included a characteristic spoke wheel patterning of vasculature on the surface of the CAM around the tumor mass, with evidence of large vessel remodeling in the periphery of the engraftment (Fig. 1A arrow). This observation was confirmed when the tumors were imaged with high-frequency ultrasound, which demonstrated dense intratumoral microvascular networks throughout the entire lesion. Blood flow in these tumors was sometimes visible on high-frequency B-mode scanning due to a combination of nucleated chicken red blood cells and a low amount of tissue attenuation. When these tumors were sampled volumetrically, as depicted in Fig. 1D, we found that the tissue was consistently hyper-vascularized throughout the entire tumor mass, with large feeding arterioles present both in the margins of the lesion and penetrating throughout the core of the tumor (Supplementary Fig. S1, Videos S1–S4). Both conventional power Doppler output from the Vevo^®^ 3100 system (Fig. 1E) and UMI images (Fig. 1F) detected arteriole flow within the tumor mass, with the UMI images also demonstrating a high degree of intratumoral vascularization. This high degree of vascularization was confirmed with fluorescent histology (Fig. 1G) as well as on H&E histology (Fig. 1B), which is consistent with Renca tumors grown in mice^24,25^.

### High-resolution UMI improved the detection of small vessel flow

**Figure 2:**
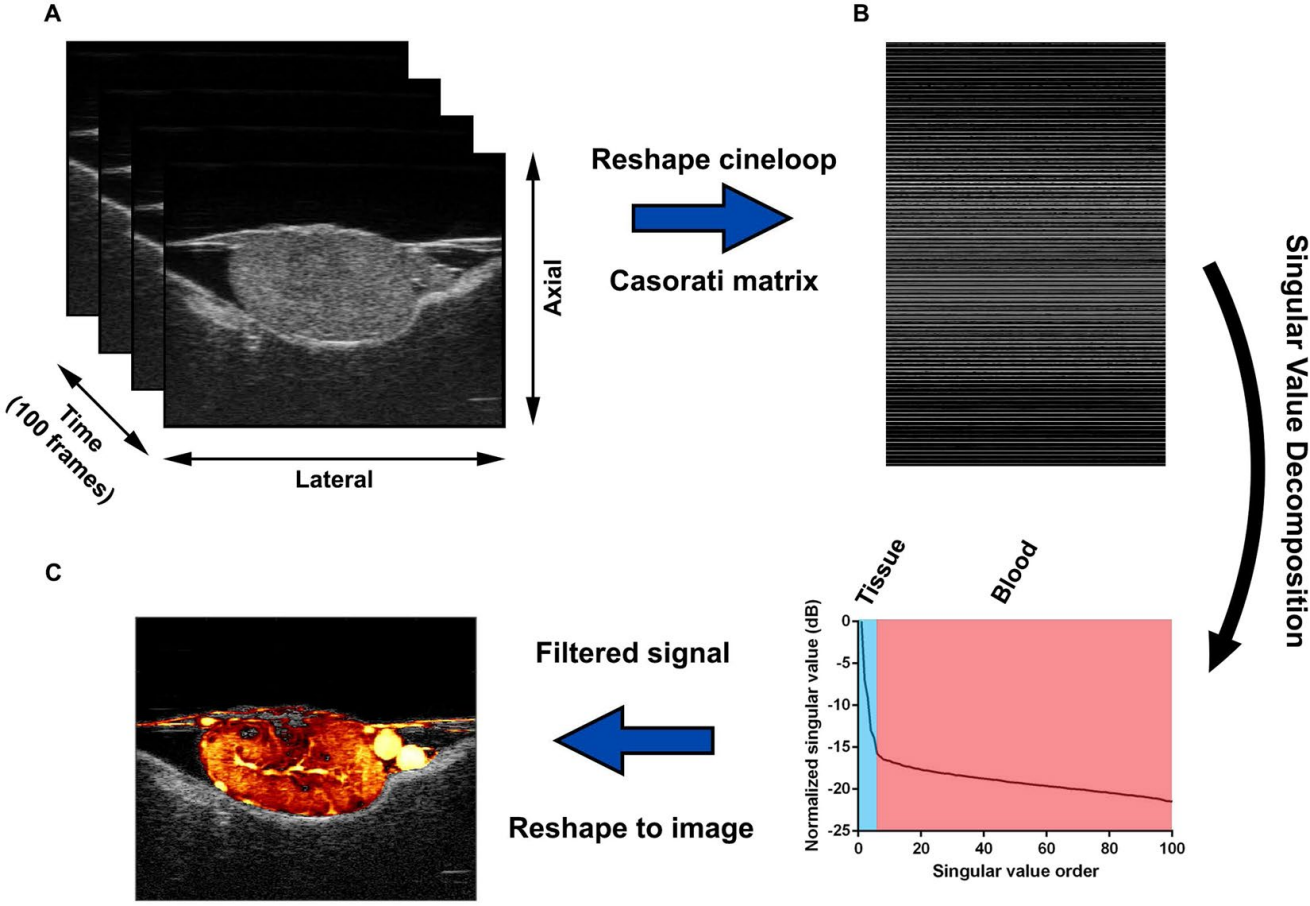
SVD-based clutter filtering of Vevo^®^ 3100 cineloops demonstrated superior microvascular detection and reduced tissue background. (**A**) B-mode IQ cineloops (100 frames) were considered as three-dimensional imaging volumes with axial, lateral, and slow-time dimensions. (**B**) These spatiotemporal data sets were reshaped into 2D Casorati matrices in a column-wise manner. Singular value decomposition of Casorati matrices decomposed the data into singular vectors and singular values, allowing for the extraction of blood signals. (**C**) An inverse SVD was performed to recover blood flow signals back to the spatiotemporal domain, resulting in a superior microvascular image in comparison to conventional power Doppler mode.

**Figure 3:**
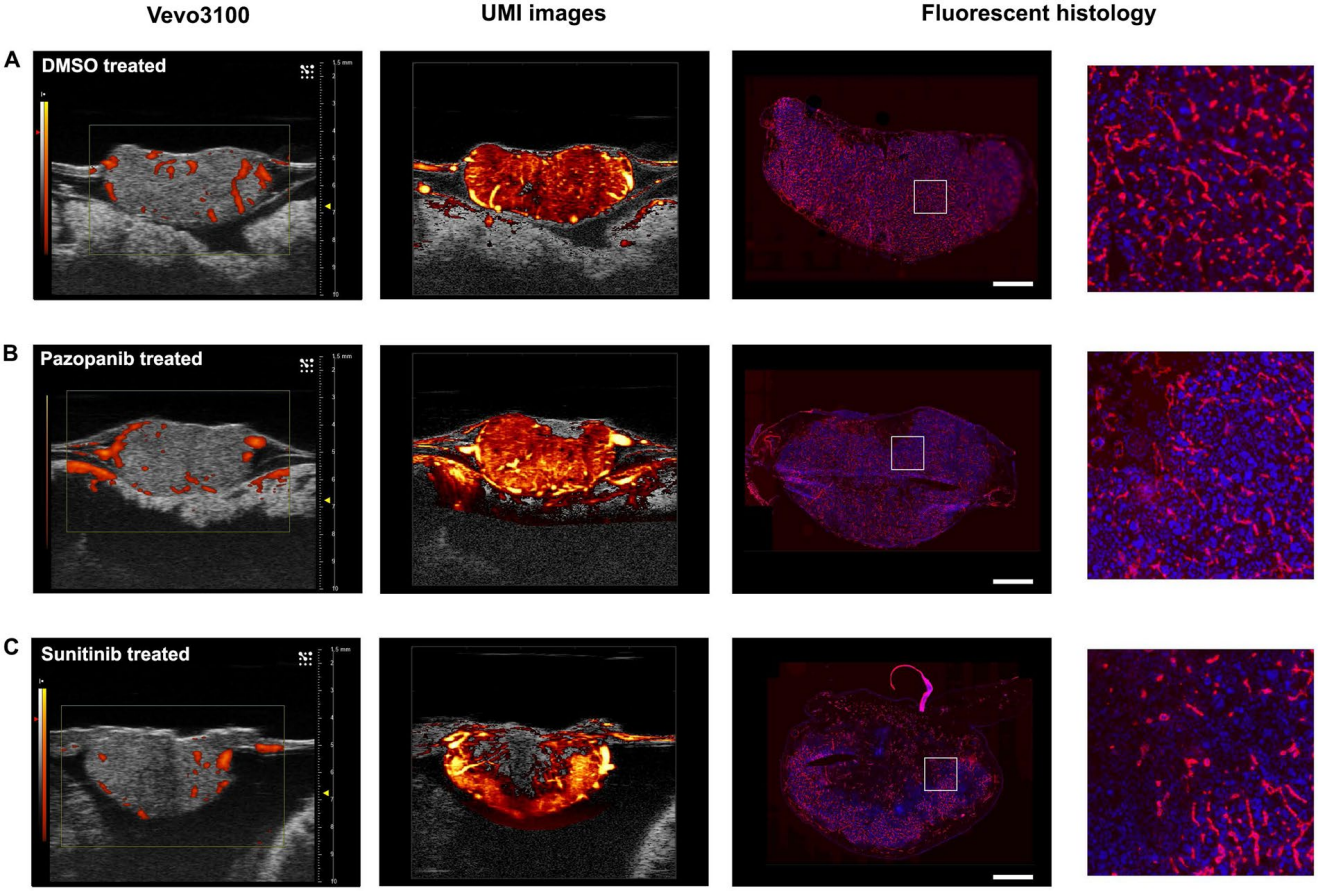
UMI improved detection and quantification of anti-angiogenic treatment effect. (**A**) Control tumor exhibited a high degree of intratumoral vascularization in conventional power Doppler imaging and UMI (Supplementary Video S2). H&E histology confirmed viable tumor cells and the presence of dense capillary networks. Fluorescent histology permitted the quantification of MFI and fluorescent vessel area as surrogate measures of microvascular density. (**B**) Conventional power Doppler imaging of pazopanib treated tumor did not reveal a significant change in vascularization. UMI image revealed localized avascular regions. Histology revealed some regional necrosis and a slightly reduced microvascular density (Supplementary Video S3). (**C**) Sunitinib treated tumor exhibited a modest decrease in tumor vascularization on Vevo^®^’s conventional Doppler image. This anti-angiogenic effect was more pronounced in UMI image (Supplementary Video S4). Histology confirmed intratumoral necrosis and a reduced microvascular density. Scale bar is 1 mm. Refer to Supplementary Fig. S1 and supplemental videos for 3D images. Rotation animations of the volumetric renderings are displayed in Supplementary Video S5.

The proposed UMI method based on SVD clutter filtering was applied to high frequency ultrasound data acquired with standard ultrasound imaging settings (see Fig. 2 and Methods section for details). The UMI images consistently demonstrated a higher degree of intratumoral vasculature (Fig. 1F, Supplementary Videos S1–S4) compared to conventional power Doppler images generated by the Vevo^®^ 3100 (Fig. 1E). Rich vascular architectures and blood flow signal in microvessels could be visualized for both control and treated tumor groups, as depicted in second column of Fig. 3 (3D images are provided in Supplementary Fig. S1 and Videos S1–S4). As Renca tumors had very dense vasculature (see the fluorescent histological images in third column of Fig. 3), blood flow signals from adjacent microvessels tended to merge together to give a ‘diffusive’ appearance on the UMI images. The spatiotemporal coherence of ultrasound signals can be used to separate fast blood flow in large vessels from slow flow in microvessels. With this approach, the major vessel tree could be separated from the ‘diffusive’ background to better show the main vascular architecture within the tumor (Supplementary Fig. S2). Although the morphology of the vessel tree may be useful for tumor characterization, our study focused on evaluation of vessel density and relative blood volume using both fast and slow blood flow signals (no separation based on flow speed was required). The observation of improved sensitivity to microvascular flow was further confirmed by images obtained from the chicken embryo brain and liver (Supplementary Fig. S3).

Renca CAM tumors imaged using the Vevo^®^ 3100 system’s power Doppler mode also demonstrated presence of intratumoral vascularization (Figs 1E, 3 first column). However, Vevo^®^’s stock Doppler mode was only sensitive to arteriole vessels and could not detect smaller vessels. In UMI images (Fig. 3, second column), the untreated control tumors showed homogeneous blood supply throughout the entire tumor volume (see Fig. 3 first row, and Supplementary Video S2), which was invisible to the system’s stock power Doppler mode. In contrast, the sunitinib treated tumors showed a more heterogeneous blood supply distribution, with a perfusion gradient starting from center of the upper tumor surface (where the drug was administrated) to periphery of the tumor. Higher level of vessel tortuosity was also present at the center region of the tumor, where the vessel density was clearly reduced (Supplementary Video S3). Again, these fine intratumoral vasculature and perfusion characteristics could not be resolved from conventional Power Doppler imaging due to lower sensitivity (Fig. 3, first column).

### Quantification of 3D tumor vessel density and relative blood volume

**Figure 4:**
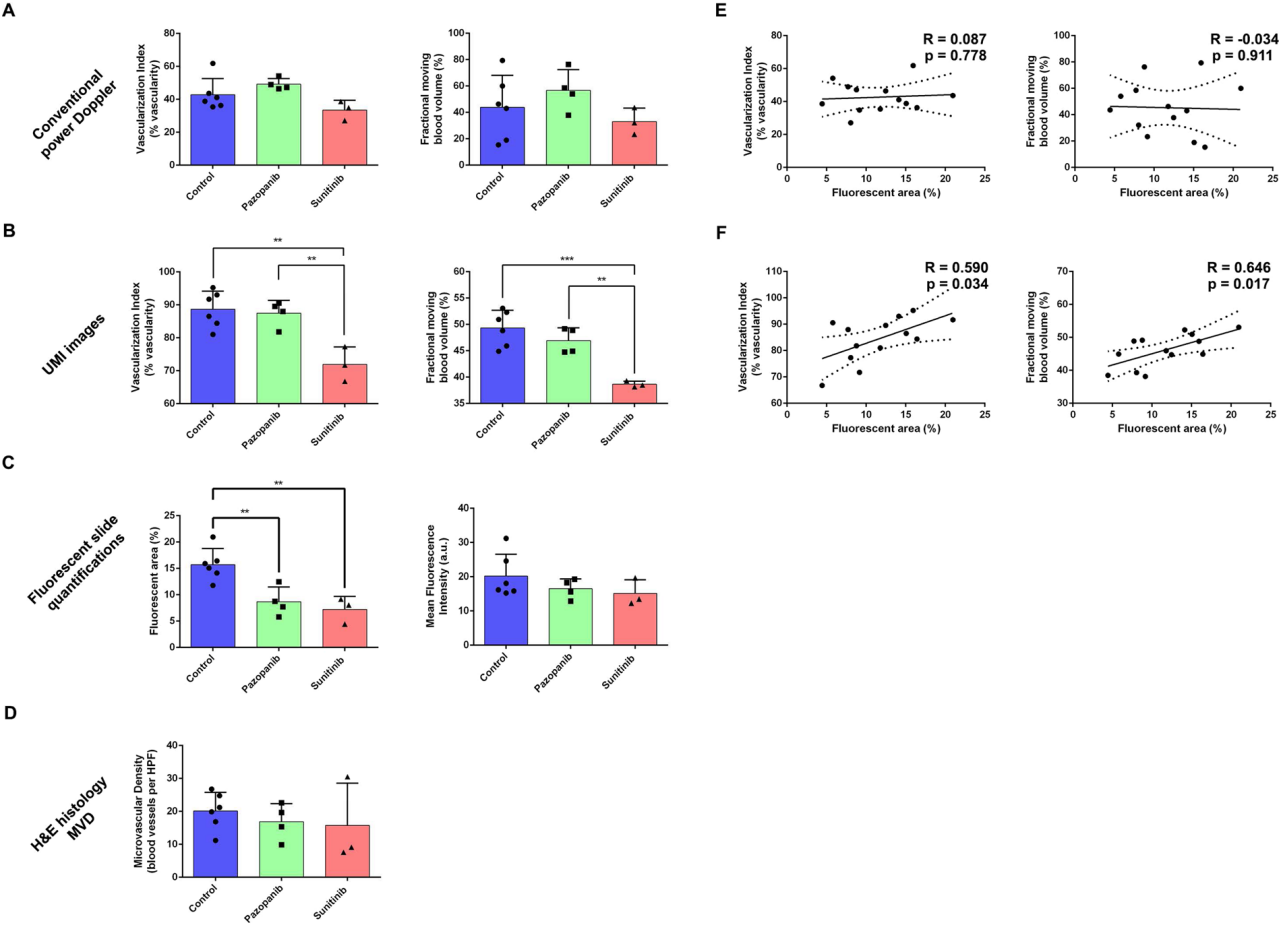
Summary statistics confirmed anti-angiogenic treatment effect. (**A**) Conventional power Doppler imaging using the Vevo^®^ 3100 system did not detect a significant decrease in intratumoral vasculature due to anti-angiogenic therapy. (**B**) The proposed UMI method based on the B-mode IQ data detected a significant decrease in vascularization index (VI) for sunitinib (multiplicity adjusted p = 0.0017) treated tumors, but not for pazopanib (p = 0.7251), in comparison to control. The confidence of this result improved when using fractional moving blood volume (FMBV) images for analysis (p = 0.0005). In both cases, pazopanib treated tumors were also found to be significantly different from the sunitinib treated cohort (p = 0.0048 for VI, and p = 0.0054 for FMBV). (**C**) Quantifications of fluorescent histology confirmed an anti-angiogenic treatment effect for both therapies (p = 0.0065 for pazopanib, and p = 0.0034 for sunitinib). (**D**) Microvascular density (MVD) evaluated from H&E slides did not demonstrate a significant therapy effect (p = 0.7364 and p = 0.6382 for pazopanib and sunitinib, respectively). (**E**) Conventional power Doppler imaging did not show a significant correlation for VI (p = 0.778) or FMBV (p = 0.911). (**F**) For UMI images, both VI and FMBV were moderately correlated with fluorescent area percentage (R = 0.590, p = 0.034 and R = 0.646, p = 0.017, respectively). All data are mean ± s.d. P-values are from a one-way ANOVA using the Holm-Sidak test to correct for multiple comparisons, and are reported as multiplicity adjusted values.

In this study, quantitative parameters including the vascularization index (VI) and fractional moving blood volume (FMBV) were used to evaluate the vessel density and relative tissue perfusion, respectively (see Methods for details). For Vevo^®^’s stock Doppler mode, neither quantitative parameters found a significant difference among the control and treatment groups through a one-way ANOVA. The mean ± s.d. for VI was 42.9 ± 9.7% for control, 49.2 ± 3.5% for pazopanib, and 33.5 ± 5.9% for sunitinib (Fig. 4A, left). For the FMBV, these values were 43.8 ± 24.3% for control, 56.7 ± 15.8% for pazopanib, and 33.0 ± 10.2% for sunitinib (Fig. 4A, right). Using UMI, a significant decrease in vascularization was detected for the sunitinib treated tumors in comparison to the controls using both VI (Fig. 4B, left, p < 0.01) and FMBV (Fig. 4B, right, p < 0.001). The mean ± s.d. for VI was 88.7 ± 5.5% for control, 87.5 ± 3.9% for pazopanib, and 71.9 ± 5.2% for sunitinib. For the FMBV, these values were 49.3 ± 3.4% for control, 46.9 ± 2.4% for pazopanib, and 38.7 ± 0.6% for sunitinib. Overall, the estimates of vascularization were higher for UMI images than those taken from the conventional Vevo^®^ power Doppler mode.

### Fluorescent histology confirmed anti-angiogenic treatment effect

To validate the robustness of the proposed ultrasensitive UMI, the above quantitative parameters were compared to the vascularization metrics obtained by histology and immunohistochemistry. Histological sections taken from rhodamine lectin-injected Renca tumors demonstrate viable cancer cell nuclei interspersed with dense vasculature (Fig. 3, right columns). DMSO treated tumors were universally vascularized throughout the entire cross-section of the histological sections (Fig. 3A), whereas pazopanib and sunitinib treated tumors exhibited regionalized decreases in capillary density near the region of topical drug dosing (Fig. 3B,C). This phenotype—of reduced vasculature around the drug dosing region—was also seen in the proposed UMI (Fig. 3, second column), but not in the conventional power Doppler mode provided by the Vevo^®^ 3100 system (Fig. 3, left column). Fluorescent area percentage, a quantitative histological metric of intratumoral vascularization, showed significant decreases in both pazopanib treated cohort (p < 0.01) and sunitinib treated cohort (p < 0.01, Fig. 4C, left). The mean ± s.d. for fluorescent area percentage was 15.7 ± 3.1% for DMSO treated tumors, 8.7 ± 2.8% for pazopanib treated tumors, and 7.2 ± 2.5% for sunitinib treated tumors. The mean fluorescent intensity of the tumor histology did not find a significant difference among the control and treatment groups (Fig. 4C, right), possibly due to variability in injection volume of rhodamine lectin before tumor excision. The mean fluorescent intensity (MFI) for each of the treatment groups was 20.2 ± 6.4 a.u. for DMSO, 16.5 ± 2.9 a.u. for pazopanib, and 15.2 ± 3.9 a.u. for sunitinib. Tumor micro vascular density (MVD), as measured on H&E sections by a pathologist (D.W.), was not found to detect a significant treatment effect between tumors, with the mean number of blood vessels per high power area being 20.1 ± 5.6 for control, 16.9 ± 5.5 for pazopanib, and 15.7 ± 12.8 for sunitinib (Fig. 4D). Tumor necrosis was not found to be significantly different between treatment groups (Supplementary Fig. S5). Tumor necrosis was less than 5% in all cases with the exception of one pazopanib treated tumor (80% necrotic) that was excluded from analysis.

### UMI was correlated with fluorescent histology

The VI and FMBV metrics derived from conventional power Doppler processing did not show a significant correlation with fluorescent area percentage, yielding a two-tailed p value of 0.778 and 0.911 respectively (Fig. 4E). For the UMI processed data, a moderate, but significant, Pearson’s correlation was found for both metrics. The correlation coefficient was R = 0.590 (p = 0.034) for VI, and R = 0. 646 (p = 0.017) for FMBV (Fig. 4F).

## Discussion

We demonstrated that high-frequency UMI is a robust method, which can be applied to IQ data from conventional ultrasound systems that acquire images using traditional line-by-line scanning, to detect and quantify tumor vascularization with marked improvement in sensitivity. High-frequency UMI outperformed conventional Doppler imaging for microvessel imaging, and detected a clear effect of anti-angiogenic therapy on the neo-vasculature of Renca tumors, which may be used as a critical metric in tumor diagnosis, prognosis, and therapy monitoring. Clinically relevant dosages of two FDA-approved anti-angiogenic agents (pazopanib and sunitinib) produced intratumoral vascular effects that were only detectible using UMI (p < 0.001 between sunitinib and control) and fluorescent histology (used as gold-standard in this study, p < 0.01 for both therapies relative to control), whereas conventional power Doppler imaging failed to detect such changes. The proposed UMI, which is based on high-frequency ultrasound imaging (50 MHz, a nominal 35 μm axial resolution), provides an imaging resolution comparable to optical technologies but with much better imaging penetration (up to 10 mm). Additionally, UMI images of other organs (brain and liver) in the chicken embryo revealed well organized microvessel structures concordant to expectations based on anatomy and physiology (Supplementary Fig. S3). This performance is not exclusive to the chicken embryo model, as pilot imaging of the kidneys of mice with a homozygous disease variant of PKD1^26^ demonstrated a characteristic vascular patterning of renal blood flow (Supplementary Fig. S4). These images increase the confidence that microvessels detected via high resolution UMI represent true blood flow, and demonstrate the feasibility of translating the UMI technique to other preclinical imaging applications. Thus the proposed UMI method offers an attractive modality to image microvessels at high sensitivity, high spatial resolution, low cost, and without ionizing radiation or the need for contrast agents.

High-frequency UMI ultrasound imaging is highly sensitive to slow blood flow signal for several reasons. Firstly, both the high ultrasound imaging frequency (50 MHz^27^) and nucleated chicken red blood cells contributed to the marked increase of backscattered ultrasound amplitude from blood in this study. Secondly, the long ensemble length of UMI (100 frames), which in this study was acquired using Vevo^®^ 3100’s B-mode raw data capture function, dramatically improved the sensitivity to blood flow in comparison to the 9-ensemble length that is used by the Vevo^®^ 3100 system’s stock power Doppler mode. Finally, the SVD filter used in the UMI method, which takes advantage of the rich spatiotemporal information provided by the long ensemble, effectively removes tissue clutter in the presence of gross tissue motion, thus enabling the detection of slower blood flow signals.

The high resolution and high sensitivity of UMI permits a more comprehensive characterization of microvasculature, enabling reliable quantification of small vessel blood flow. The measured VI and FMBV—which reflect blood vessel density and relative tissue perfusion respectively—were shown to correlate well with the fluorescent histological reference standard. In comparison, conventional power Doppler imaging was less sensitive to the anti-angiogenic treatment effect seen in this study. Therefore, UMI could serve as a surrogate imaging-based biomarker for histological measures of tumor vascularization that is both non-destructive and easy to implement in 3D, permitting longitudinal tumor monitoring of anti-angiogenic therapies. Generally, VI tended to overestimate tumor microvascular density, since the binary threshold does not take into consideration the partial volume effect of a large sampling volume pixel size relative to the microvascular lumen diameter. FMBV, however, explicitly considers the impact of fractional blood volume weighting and allows for the calibration of tissue perfusion evaluation—a critical benefit for highly vascularized tissues such as Renca tumors.

A limitation of this study is that only power Doppler images were obtained, and consequently neither pulse-wave Doppler nor color-flow imaging was performed. Color flow images are theoretically obtainable from the dataset, but it lacks sensitivity as compared with power Doppler images and the highest measurable flow speed is limited by the effective framerate of acquired IQ ultrasound data. Alternatively, directional power Doppler imaging, as shown in Supplementary Fig. S6, can be incorporated into the proposed high-frequency UMI to provide information on the flow direction and vascular morphology, which may be particularly helpful for better vessel separation and characterization. A further limitation of high-frequency UMI is the required acquisition of a relatively long ensemble of ultrasound data (100 frames at 22 Hz in this study), which restricts the capability for real-time imaging and requires that physiological motion is minimized. The requirement for a 100 frame ensemble can be relaxed somewhat as visually comparable UMI images can be generated with only 40 frames of acquisition, as shown in Supplementary Fig. S7. Conventional Doppler mode is still necessary to guide the imaging volume and identify target tissues, with UMI being implemented in a post-processing step to achieve detailed vascular characterization. A fast SVD clutter filter technique was recently proposed to address the computational challenge of the UMI method^20^, permitting a potential real-time implementation of high-frequency UMI imaging for 2D acquisitions. Translating the technique into real-time 3D imaging would prove to be more problematic given the technical limitations of the linear translation motor used to sample the tumor in three-dimensions.

High-frequency UMI imaging improves the sensitivity of blood vessel detection without the need for contrast agents, but does not improve the imaging resolution beyond the diffraction limit of the ultrasound wave frequency. As such, it is necessarily bounded by the inherent trade-off between imaging resolution and imaging depth. This trade-off limits the potential clinical translation of the UMI technique for microvascular imaging except in the specific clinical imaging scenarios where high-frequency imaging resolution is preferred and/or a shallow imaging depth is acceptable (e.g.: superficial imaging targets such as lymph nodes). This trade-off is circumvented by state-of-the-art super-resolution microbubble imaging techniques^28–30^, which permit the localization of microvasculature at clinical relevant penetration depths by exploiting the acoustic point scattering properties of spatially isolated microbubbles. However, such techniques greatly increase the experimental and computational complexity of microvascular imaging and cannot be considered as truly non-invasive as they require intravascular injection of contrast agents. A direct comparison between UMI and super-resolution microbubble imaging is outside of the scope of this manuscript.

An interesting observation from our histological analysis (Fig. 4) was that fluorescent slide quantifications (Fig. 4C) demonstrated a clear treatment effect for pazopanib and sunitinib treated tumors in comparison to the control group, but this treatment effect was not evident on H&E MVD quantifications (Fig. 4D). A plausible explanation for this divergent result could be due to the different volumetric sampling of the sections and/or the inherent variability of hotspot-based microvessel density quantification. Fluorescent quantification was algorithmic, and could be applied to the entire section uniformly, whereas H&E MVD counts were performed by an expert pathologist on isolated regions of high vessel concentration. Furthermore, rhodamine lectin, used to fluorescently label chick vasculature in this study, binds to endothelial glycoproteins in the luminal space of actively perfused vasculature. By contrast, the evaluation of MVD on H&E histological sections does not consider whether or not the counted vessels are functionally active. Given that MVD counts are a controversial biomarker for anti-angiogenic therapy response^31^, we are hesitant to conclude anything definitive from these differences in histological quantification. In addition, the focus of this manuscript is concerned with ultrasound signal processing and not explicitly the study of treatment efficacy associated with sunitinib or pazopanib. Meanwhile the sample size of our study is small and may be limited for elucidating the exact reason for this difference in fluorescent versus H&E based histological quantification.

This proof of concept study has demonstrated the feasibility of high-frequency UMI based on SVD clutter filtering and IQ data acquired from a commercial preclinical ultrasound system. The presented method does not require administration of contrast agents and is compatible with commercial preclinical ultrasound scanners without the need of software or hardware modifications. The results of this study support the utility of UMI in preclinical anti-angiogenic drug evaluation, and this widely accessible quantitative imaging tool could be beneficial if and when implemented in other vascular imaging scenarios such as ischemia assessment and functional tissue monitoring.

## Methods

### Ethical approval

No IACUC approval was necessary to perform the chicken embryo experiments presented in this manuscript, since avian embryos are not considered to be live vertebrate animals according to the NIH PHS policy. All mouse imaging demonstrated in the supplemental data was approved under an Institutional Animal Care and Use Committee protocol (IACUC protocol number A44314).

### Preparation of reagents

Stock solutions of sunitinib and pazopanib were obtained from LC Laboratories (Woburn, MA) and dissolved with dimethyl sulfoxide (DMSO). Stock solutions were diluted with phosphate buffered saline (PBS) to achieve a final concentration of 10 µM for sunitinib (to achieve a targeted intratumoral concentration of 10 µmol/L^32^), and 150 µM for pazopanib (pazopanib is clinically dosed at 800 mg daily in comparison to sunitinib at 50 mg daily). Vehicle control was 1% DMSO diluted in PBS. Rhodamine lectin was diluted at a 1:10 ratio with PBS immediately prior to intravascular injection. Stock Hoechst solution was diluted at a 1:1000 ratio with PBS.

### Cell culture

The Renca cell line was obtained from the American Type Culture Collection Inc. (Bethesda, MD) and maintained in RPMI 1640 media (Wisent, QC) supplemented with 10% FBS (Hyclone, UT), non-essential amino acids (NEAA) (0.1 mM), sodium pyruvate (1 mM), and L-glutamine (2 mM). Cells were sub-cultivated when above 80% confluency at a 1:5 ratio. All cells were kept in a 37 °C, 5% CO_2_ humidified incubator.

### Engraftment of cell lines into the CAM

Cell lines were grown to >80% confluency and trypsinized (0.05% Trypsin-EDTA, Wisent) on the day of engraftment. Detached cells were transferred into 15 mL falcon tubes and centrifuged for 5 minutes at 300 g. The cell pellet was re-suspended in 10 mL of PBS, and a 10 µL sample of the cell suspension was analyzed using the Countess cell counter (Life Technologies) to get a cell count. Cells were then centrifuged again for 5 minutes at 300 g. Matrigel (BD Bioscience) was mixed with the cell pellet to obtain a final mixture of 5 × 10^5^ cells in a 10 µL inoculation dose. The mixture of cells with Matrigel was kept on ice until implantation into the CAM.

Fertilized chicken eggs were obtained from Hoover’s Hatchery (Rudd, IA), and transferred into a plastic weight boat on the fourth day of embryonic development (EDD-4). On EDD-9, a 5-mm disk of autoclaved Whatman No.1 filter paper was used to score the chorioallantoic membrane, and 10 µL of the cell mixture was added into the abrasion. A total of 24 chicken embryos were inoculated with Renca cell line tumors. Topical dosage (5 µL) of anti-angiogenic agents (sunitinib, pazopanib, or control) were administrated daily, beginning on EDD-11 and continuing until EDD-18 (day of ultrasound imaging). Tumor bearing embryos were split evenly into each treatment group on EDD-11.

### Ultrasound imaging protocol

Ultrasound imaging was performed using a Vevo^®^ 3100 high-frequency imaging system (FUJIFILM VisualSonics Inc., Toronto, Canada) equipped with a 50 MHz linear array transducer (MX700, 35 μm nominal axial resolution, 70 μm nominal lateral resolution) transmitting in RF-power Doppler mode. Coupling gel (Aquasonic^®^ 100, Parker Laboratories, Inc., Fairfield, NJ) was applied over the surface of the transducer to provide an acoustic standoff and adequate coupling to the CAM tumor surface. The MX700 transducer was attached to the commercially available linear stepper motor (P/N 11484, VisualSonics Inc.) within the Vevo® integrated rail system and positioned above the center of the tumor mass. The imaging field of view was set to 10.00 mm (axial) by 9.73 mm (lateral), with a Doppler color box positioned around the tumor cross-section, resulting in a frame rate of 22 Hz. Operator settings were fixed to a PRF of 2 kHz, a gate of 2, sensitivity of 1, and a ‘low’ wall filter. B-mode acquisition was done using 100% transmit power and 1 transmit cycle, power Doppler acquisition was done with 100% transmit power and 2 transmit cycles. Using the system stepper motor controls, the transducer was incrementally stepped, in a stop and go fashion, through the tumor volume by 0.25 mm per step (Fig. 1D). An in-phase/quadrature (IQ) cineloop of 100 B-mode and power Doppler frames was acquired for each step through the tumor volume after allowing for any tissue motion from the motor translation to be minimized.

### Ultrasound microvessel imaging (UMI)

The IQ data acquired in power Doppler mode includes both B-mode IQ data and Doppler IQ data (9 ensembles) in an interleaved manner (i.e. a frame of B-mode IQ data was acquired following each Doppler frame consisting of 9 ensembles), as shown in Supplementary Fig. S2A. Either the B-mode or Doppler packet IQ data can be used separately for UMI (see Supplemental Material). Unless otherwise stated, the UMI was performed based on the B-mode IQ data in this study. The spatiotemporal SVD-based clutter filtering was applied on the “synthetic” long ensemble data set (100 ensembles) which was constructed using the B-mode IQ data, to extract the blood flow signals. Specifically, consider the B-mode IQ data as ${S}_{0}(x,y,t)$ with a size of ${N}_{z}\times {N}_{x}\times {N}_{t}$, where ${N}_{z}$ and ${N}_{x}$ correspond to the data size in axial and lateral dimensions respectively, and ${N}_{t}$ corresponds to the slow-time dimension (i.e., the ensemble size). The spatiotemporal data set ${S}_{0}(x,y,t)$ is first reshaped into a 2D Casorati matrix with dimensions of ${N}_{z}\times {N}_{x}$ by ${N}_{t}$, as depicted in Fig. 2. The SVD of this 2D Casorati matrix decomposes the data into singular vectors and singular values. Tissue signal typically has high speckle strength and spatiotemporal coherence and is represented by the low-order, larger singular values in SVD. The corresponding tissue signals are suppressed by forcing the lower-order singular values to zero. This singular value threshold cutoff value is automatically determined by the decay rate of the singular value curve^19^. In most imaging applications, a second, high-order, threshold must also be set to eliminate noise, as detailed in^19^. However, the tissue attenuation of the chicken embryo tumor model is minimal, and the line-by-line focused imaging used in this study had a sufficiently high signal-to-noise ratio (SNR) to make the noise suppression unnecessary in this application. Then an inverse SVD was performed to derive the spatiotemporal blood flow signals ${S}_{B}(x,y,t)$. The microvessel power Doppler image is then obtained by cumulating the power of the blood flow signals along temporal dimension and displayed in decibel scale, as shown in Fig. 1F. The SVD based clutter filtering was performed separately for every plane of the tumor and 3D volumetric microvessel images can be reconstructed from the multiple planes of 2D images for each tumor. (See Supplemental Material). Noise suppression can be improved using the relatively higher PRF power Doppler ultrasound IQ data (see Supplemental Material).

### Ultrasound quantification index

Two quantitative metrics, vascularization index (VI) and fractional moving blood volume (FMBV), were derived from the microvessel power Doppler images of the tumor. The tumor region-of-interest (ROI) was first manually delineated based on both the B-mode and microvessel images for each 2D plane of the tumor. VI is defined as the area of the blood vessel within the ROI divided by the total area of the tumor ROI, as:

1 $$VI=\frac{{A}_{Vessel}}{{A}_{tumor}},$$

where ${A}_{Vessel}$ is the vessel area, calculated as the area above a manually-selected threshold for the power Doppler image, and ${A}_{tumor}$ is the area of the ROI. VI presents a measurement of the vessel density of the tumor.

For FMBV measurements, we followed the classic method introduced by Rubin *et al*.^33^ to access the tissue perfusion. Briefly, pixels that represent 100% blood (i.e., pixels that are completely located inside a major vessel) were used as the normalization factor to calculate fractional blood volume of pixels that contain both blood and tissue (e.g., pixels on the edge of a large vessel or pixels containing microvessels smaller than the pixel size). In this study, FMBV is measured based on the logarithmic display of the normalized power Doppler image (dB scale), where 0 dB indicates the maximum intensity value of the Power Doppler image. Power Doppler pixels with intensity values above −6 dB were considered as pixels with 100% FMBV, and power Doppler pixels with intensity values between −50 dB and −6 dB were linearly scaled to represent FMBV ranging from 0% to 100%. The final FMBV is obtained by summation of the FMBV value of each pixel over the ROI, and normalized by the total number of pixels within the ROI.

### Histological sectioning

Following ultrasound imaging, the vasculature of tumor bearing chicken embryos was injected with 70 µL of rhodamine lectin (*lens culinaris* agglutinin^34^) which binds to the endothelial cell surface of actively perfused vasculature. The lectin was circulated for 20 minutes, and then the tumors were excised and embedded into OCT (optimal cutting temperature) blocks for cyrosectioning. Tumor blocks were sectioned at a thickness of 10 µm. Sections were stained either with Hematoxylin and Eosin (H&E) for white-light imaging, or with Hoechst for fluorescent imaging.

### Histological analysis

H&E histological sections were reviewed by a board-certified pathologist (D.W.) to evaluate tumor microvascular density (MVD). Blood vessels lined by endothelial cells were counted on cross-sections at 400x magnification. Twenty high power fields were counted for each sample and the mean number per high power field was calculated. The necrotic area was estimated on each section and averaged on three sections per sample.

Fluorescent histological sections were digitized using a Zeiss Axio Scan.Z1 with a CY3 channel exposure of 400 ms, and a DAPI channel exposure of 3 ms. CZI files were exported as tiff images and analyzed in ImageJ. Manual segmentations of the tumor cross-sections were used to quantify the mean fluorescent intensity (MFI) of the rhodamine lectin stain as a metric of intratumoral vasculature. The images were then binarized using Otsu’s method threshold and a measure of the number of ‘vessel’ pixels in the images were calculated as a percentage of the total number of pixels in the imaging cross-section. This metric was termed the fluorescent area percentage, which served as a surrogate measure for microvascular density.

### Statistical analyses

Ultrasound quantifications were summarized by averaging the imaging planes across the entire sampled volume (Fig. 1D). Histological quantifications were averaged across two slides, with three sections per slide. Statistical significance was determined via a one-way analysis of variance (ANOVA) with an alpha threshold of 0.05. Multiple comparisons were corrected for via the Holm-Sidak test.

## Supplementary information

Supplemental Materials

Video S1

Video S2

Video S3

Video S4

Video S5

## Data Availability

The data that support the findings of this study and the codes for processing the raw IQ data obtained from Vevo^®^ 3100 ultrasound imaging system are available from the corresponding authors on request.
