# Supplementary material for: Noninvasive Contrast-Free 3D Evaluation of Tumor Angiogenesis with Ultrasensitive Ultrasound Microvessel Imaging
Source: Sci Rep. 2019 Mar 20;9:4907. doi: 10.1038/s41598-019-41373-0 (PMC6426859; doi:10.1038/s41598-019-41373-0)
Supplement: Supplementary file 1 — Supplemental Materials [file 41598_2019_41373_MOESM1_ESM.pdf]

Noninvasive Contrast-Free 3D Evaluation of Tumor Angiogenesis with Ultrasensitive Ultrasound Microvessel Imaging

Chengwu Huang[1,§], Matthew R. Lowerison[2,§], Fabrice Lucien[2], Ping Gong[1], Diping Wang[3], Pengfei Song[1,*], Shigao Chen[1,*]

[1] Department of Radiology, Mayo Clinic College of Medicine and Science, Mayo Clinic, Rochester, MN
[2] Department of Urology, Mayo Clinic College of Medicine and Science, Mayo Clinic, Rochester, MN
[3] Department of Laboratory Medicine and Pathology, Mayo Clinic College of Medicine and Science, Mayo Clinic, Rochester, MN
[§] These authors contributed equally to this work.

*Correspondence and requests should be addressed to either P.S. or to S.C.:

Pengfei Song, Ph.D.
Department of Radiology
Mayo Clinic
200 First Street SW
Rochester, Minnesota 55905
USA
Email: song.pengfei@mayo.edu

Shigao Chen, Ph.D.
Department of Radiology
Mayo Clinic
200 First Street SW
Rochester, Minnesota 55905
USA
Email: chen.shigao@mayo.edu

3D ultrasound microvessel imaging

Tumors were scanned plane-by-plane with an inter-plane step size of 0.25 mm (**Fig. 1D**), requiring approximately 20-30 separate planes to cover the entire tumor volume. **Supplementary Figure S1** shows three different imaging planes, and the final 3D reconstruction of the microvessel images, for each of the three tumors shown in **Fig. 3** (DMSO treated, pazopanib treated, and sunitinib treated, respectively). Corresponding videos of 3D volumetric images can be found in **Supplementary Videos S2 – S4**, respectively.

Multi-scale microvessel analysis

The UMI processing was based on ultrasound IQ data acquired using the standard power Doppler imaging settings, which includes the acquisition of low frame rate B-mode data and high frame rate power Doppler packet in an interleaving manner, as shown in **Supplementary Fig. S2A**. More specifically, a frame of B-mode IQ data was acquired following each packet of Doppler data, and for the current study, each acquisition included 100 frame of B-mode data and 100 packets of Doppler data. Each packet contained 9 ensembles of data with a high frame rate of 2kHz, and the B-mode frame rate was around 20 Hz. Either the low frame rate B-mode data or the high frame rate Doppler packets could be used for UMI. The advantage of utilizing B-mode data was a lower data footprint and therefore a lower processing cost, which was done for most of the data processing in this study. However, for multi-scale microvessel analysis, the high frame rate Doppler data was used (all packets were combined to produce a long ensemble data set, as shown in **Supplementary Fig. S2A**). The fast flow and slow flow signals can be further separated following tissue clutter rejection (SVD filtering) by leveraging the high frame rate data to differentiate signals with different spatiotemporal coherences, as depicted in **Supplementary Fig. S2B.** Faster blood flow de-correlates faster than slow flow in spatiotemporal domain, and therefore tends to reside in the high-order singular values, as

opposed to slow-flow small vessels which tend to reside in the low-to-medium-order singular values. Preserving only low-to-medium-order singular values (from 25 to 40, **Supplementary Fig. S2B**, second column), which represent slow flow signals at the level of perfusion, produces a 'diffusive' appearance in UMI images from highly-vascularized tumors and may reveal the distribution of intratumoral perfusion. When using the higher-order singular values (from 110 to 900, **Supplementary Fig. S2B**, third column) to reconstruct UMI images, the 'diffusive' slow flow signals are suppressed, and the major vascular architectures with faster blood flow can be more clearly shown. By selecting different segments of singular values on the singular value curve, one can therefore reveal microvessels at different sizes and flow speeds. Such multi-scale microvessel analysis may be beneficial for evaluating treatment effects on different levels of vessels. A further advantage of using high frame rate Doppler data is that the background noise (i.e., electronic noise), which manifests in the form of "ramp-shaped" appearance in the background (**Supplementary Fig. S2B**, third column) of the power Doppler images with low signal-to-noise-ratio (SNR), can be suppressed by using a noise profile derived from a lag-one temporal autocorrelation of the ensembles (as shown in **Supplementary Fig. S2B**, second row). The principle behind this noise suppression method is that electronic noise between adjacent ultrasound data frames is uncorrelated, and thus can be effectively suppressed by lag-one autocorrelation.

Application to different tissue types

In order to demonstrate the feasibility of the proposed method to other tissue types, the brain and liver of the chick embryo were scanned with the same standard imaging settings used in the paper. **Supplementary Figure S3** shows the conventional power Doppler images obtained from the ultrasound system (left column) and the corresponding ultrasensitive UMI images (right column). Since penetration was limited by the chick embryo skull bone and soft tissue

attenuation of high-frequency ultrasound, high frame rate Doppler ultrasound data were used to derive microvessel images for the purpose of noise suppression under the low SNR conditions as described above. The proposed ultrasensitive UMI successfully captured the tiny and organized vessel structure in the brain and liver (**Supplementary Fig. S3**, right column), which could not be resolved by the conventional power Doppler imaging of the system (**Supplementary Fig. S3**, left column).

Mouse kidney imaging

All mouse imaging presented here was approved under an Institutional Animal Care and Use Committee protocol (IACUC protocol number A44314). Animals were anesthetized using 3% isoflurane mixed with medical oxygen for induction, and 2% isoflurane mixed with oxygen supplied using an anesthesia mask for maintenance. The mouse was secured to a heated physiological monitoring platform (THM-150, VisualSonics Inc.) and imaged using a 40MHz transducer (MX550S) transmitting in IQ B-mode. The transducer was positioned to produce an anatomically transverse imaging plane of the right kidney. The imaging field of view was reduced to 6.08 mm laterally and a single transmit focus resulting in a frame-rate of 232 Hz. An IQ cineloop of 100 frames was acquired for offline processing. UMI imaging revealed a characteristic renal blood vessel patterning from the outline kidney cross-section (**Supplementary Fig. S4**)

Directional power Doppler imaging

Ultrasound power Doppler imaging presents the backscattered signal power from moving red blood cells and does not include information on blood flow speed or flow direction. Ultrasound color flow imaging provides flow speed and flow direction information, but suffers from low

sensitivity to slow flows, aliasing, and tissue motion-induced artifacts [1]. Directional power Doppler imaging provides a tradeoff between power Doppler and color flow, which can reveal the direction of the blood flow while preserving the sensitivity of power Doppler imaging (**Supplementary Fig. S6**). Directional power Doppler images were obtained by separating the clutter-filtered blood flow signal $S_B(x,y,t)$ into positive frequency components and negative frequency components for each imaging pixel. Specifically, a fast Fourier transform of $S_B(x,y,t)$ was performed along temporal direction, and the power of the positive and negative spectrum was calculated, respectively, to obtained two images with opposite flow directions[1]. The two images were then color-coded with red color representing flow towards transducer and blue color representing flow moving away from the transducer, as shown in **Supplementary Fig. S6B**. Flow direction may provide useful information in addition to UMI power Doppler images for vascular characterization, especiallly in applications where distinguishing between arterial and venous flow in opposite directions is necessary.

References:

1. Mace, E. *et al.* Functional ultrasound imaging of the brain: theory and basic principles. *IEEE Trans Ultrason Ferroelectr Freq Control* **60,** 492–506 (2013).

Supplementary Figure Legends

Figure S1 **|** *Volumetric UMI imaging*

Example 2D image planes and the corresponding 3D reconstruction of microvessel images for the (**A**) DMSO treated, (**B**) pazopanib treated, and (**C**) sunitinib treated tumors shown **Fig. 3** (3D volumetric images are provided by **Supplementary Videos S2 – S4**).

Figure S2 **|** *Multi-scale UMI microvessel analysis*

Multi-scale microvessel analysis and noise suppression based on high frame rate ultrasound data. (**A**) The standard power Doppler mode includes the generation of B-mode data and Doppler packet data in an interleaving manner. For multi-scale microvessel analysis, only the high frame rate Doppler data are used. (**B**) Examples of multi-scale microvessel images. By selecting different segments of singular values on the singular value curve, microvessels at different sizes and flow speeds can be revealed (upper row). By calculating the temporally lag-one autocorrelation, instead of the conventional lag-zero autocorrelation (signal power) of the blood flow signals, the background noise floor can be largely suppressed and UMI SNR could be improved (corresponding multi-scale images at bottom row).

Figure S3 **|** *UMI images of chick anatomy*

Conventional power Doppler images taken from the Vevo® 3100 system and the proposed ultrasensitive microvessel images obtained from (**A**) brain and (**B**) liver of the chick embryo.

Figure S4 **|** *UMI image of mouse kidney*

Ultrasensitive microvessel imaging of a mouse kidney (outlined in cyan) acquired from an anatomically transverse imaging plane revealed characteristic renal vascular patterning.

Figure S5 | H&E stained tumor section of therapy treated Renca tumors

High-power fields of CAM engrafted Renca tumors reveal viable cancer cells and the presence of a high degree on intratumoral vasculature. The overall tumor viability and necrosis was not significantly different between control, sunitinib, and pazopanib treated tumors.

Figure S6 | Directional power Doppler imaging

(**A**) Original microvessel power Doppler image of a DMSO treated tumor obtained with the proposed UMI. (**B**) Corresponding color-coded directional power Doppler image with red color representing the motion towards transducer and blue color away from the transducer.

Figure S7 | Effect of number of frames on UMI quality

UMI images generated using a decreasing number of frames at a fixed frame rate. A UMI image generated with 40 frames can provide a visually comparable image quality to a UMI image generated using 100 frames. The UMI performance begins to deteriorate significantly when the number of frames is reduced down to 20 frames.

Supplementary Video Legends

***Video S1* |** Volumetric UMI imaging of the DMSO-treated tumor shown in **Fig. 1E-1G**

***Video S2* |** Volumetric UMI imaging of the DMSO-treated tumor shown in **Fig. 3A**

***Video S3* |** Volumetric UMI imaging of the pazopanib-treated tumor shown in **Fig. 3B**

***Video S4* |** Volumetric UMI imaging of the sunitinib-treated tumor shown in **Fig. 3C**

***Video S5* |** Rotation animations of volumetric UMI images shown in **Fig. S1** and **Videos S2-S4**

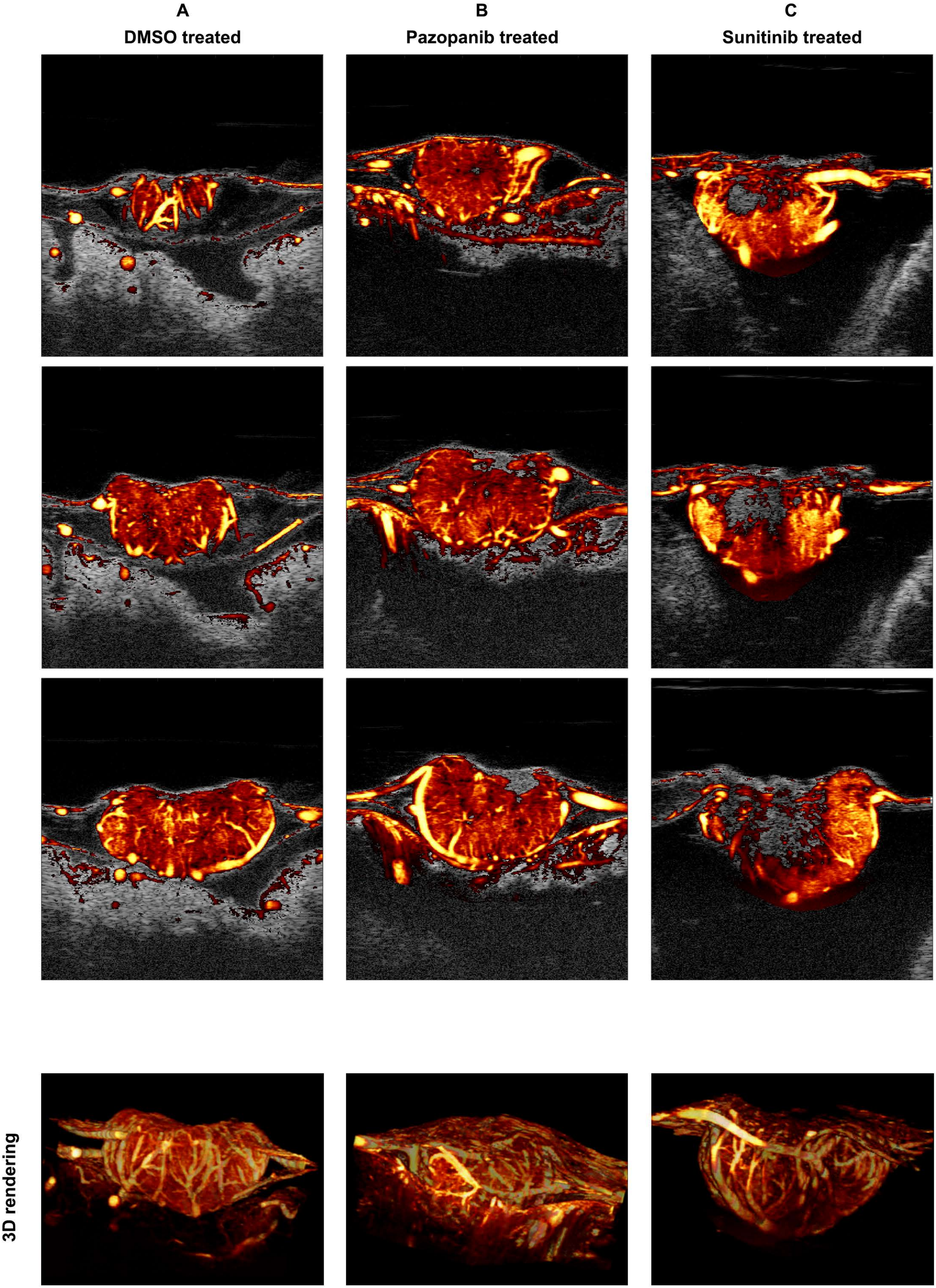

A
DMSO treated
B
Pazopanib treated
C
Sunitinib treated
3D rendering

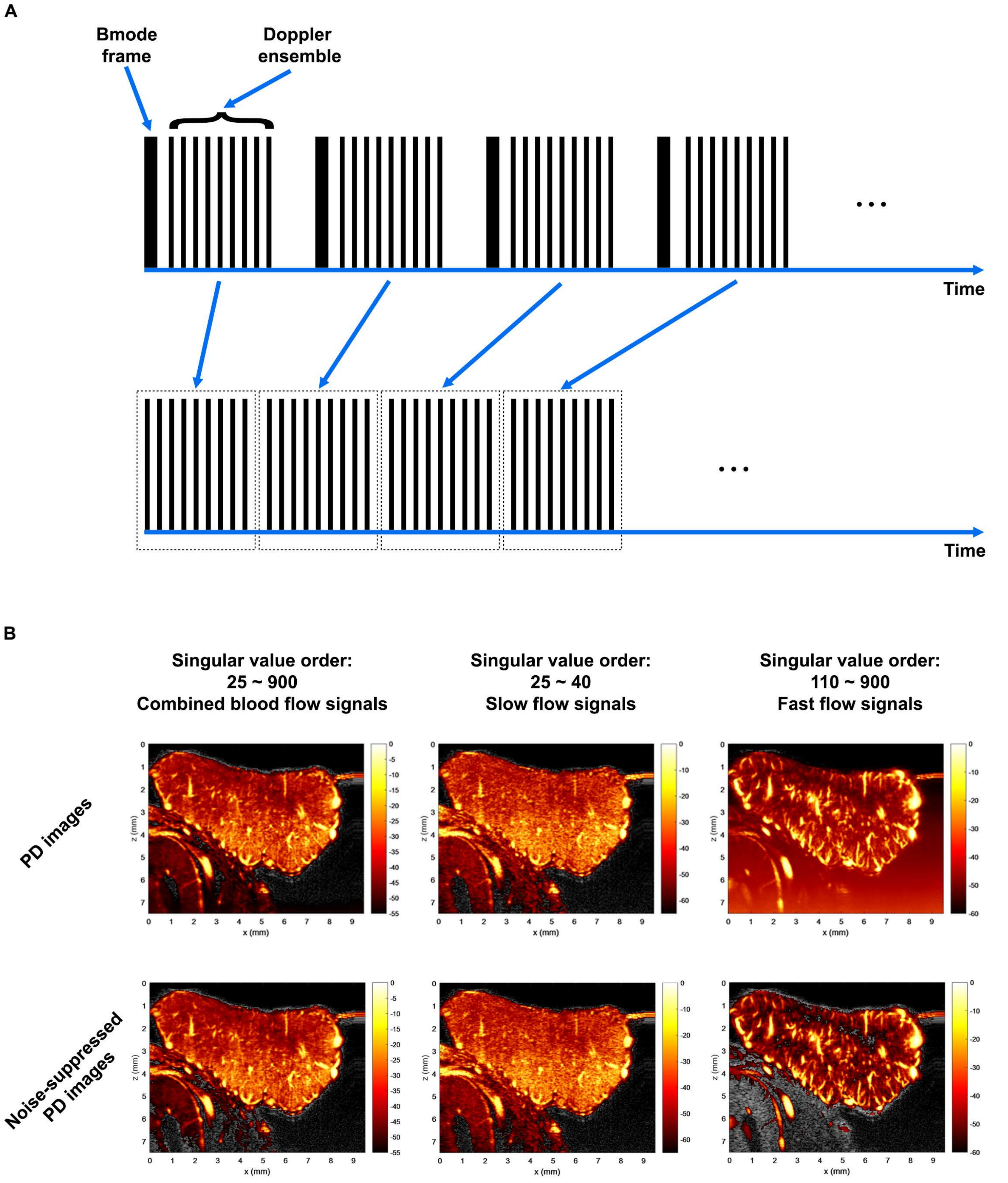
A
Bmode frame
Doppler ensemble
Time
Time
B
Singular value order: 25 ~ 900 Combined blood flow signals
Singular value order: 25 ~ 40 Slow flow signals
Singular value order: 110 ~ 900 Fast flow signals
PD images
Noise-suppressed PD images
z (mm)
x (mm)

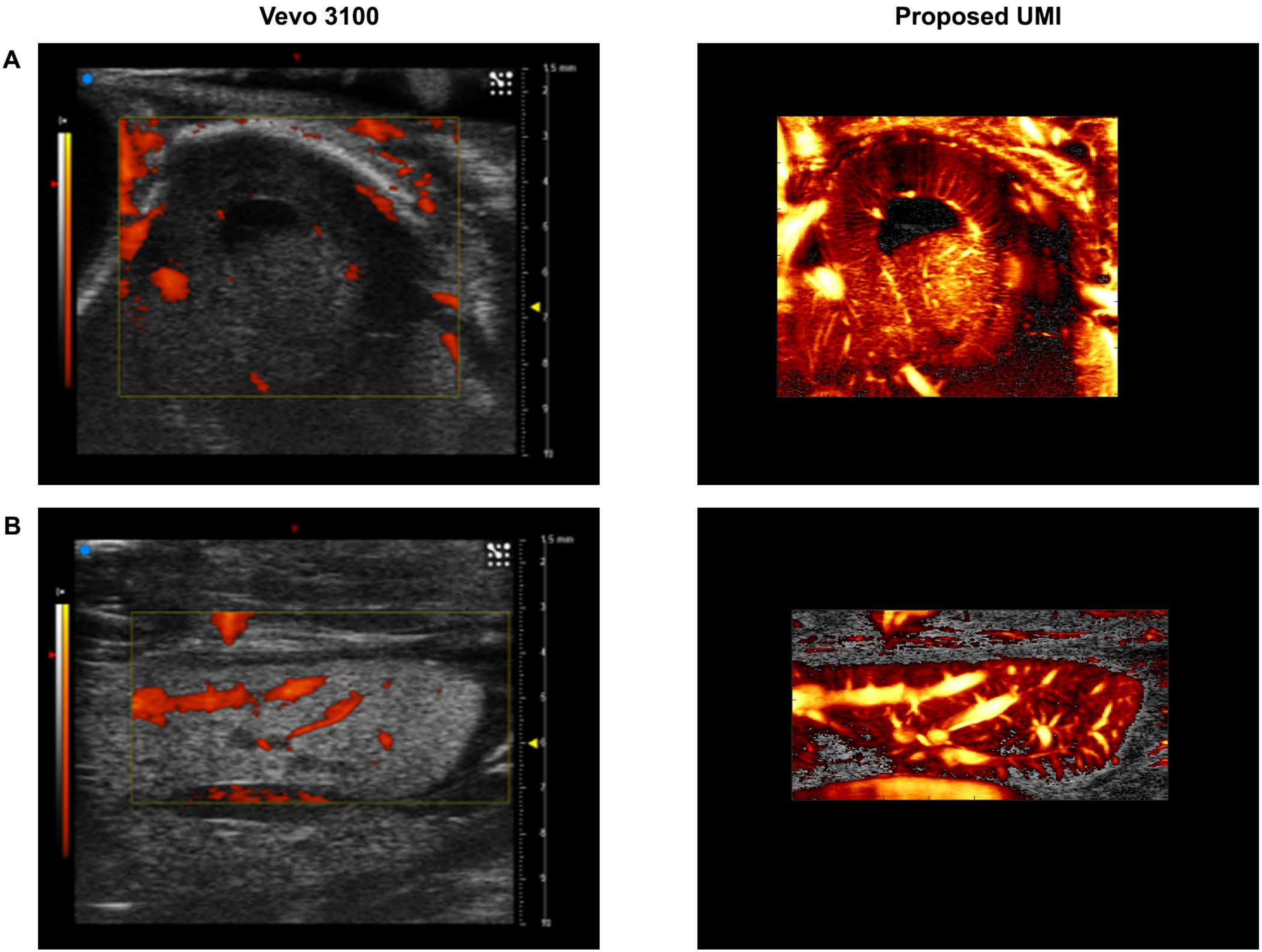
Vevo 3100
Proposed UMI
A
B

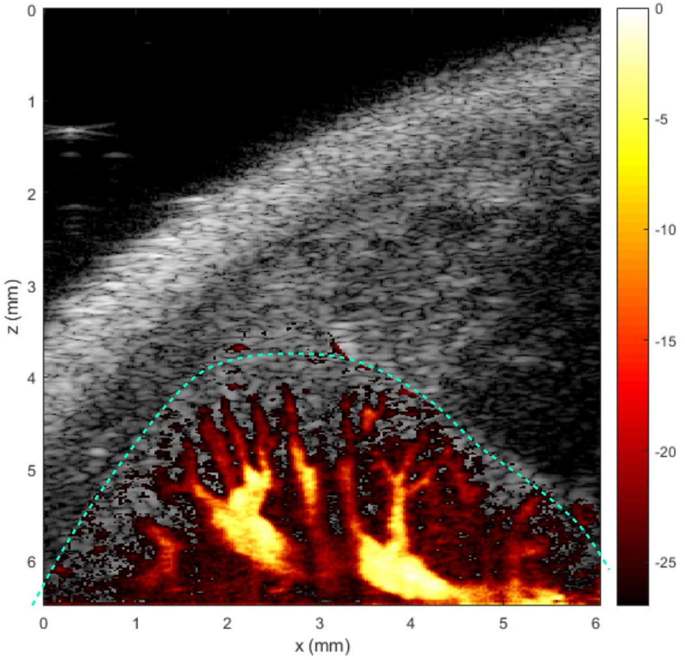

z (mm)
x (mm)
0
1
2
3
4
5
6
0
-5
-10
-15
-20
-25

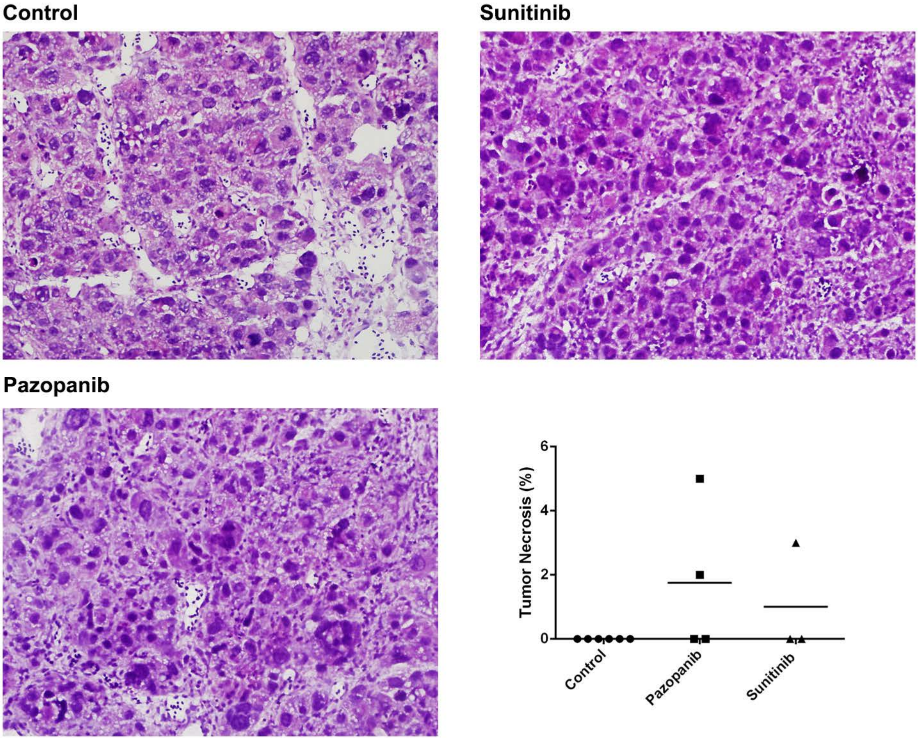
Control
Sunitinib
Pazopanib
Tumor Necrosis (%)
6
4
2
0
Control
Pazopanib
Sunitinib

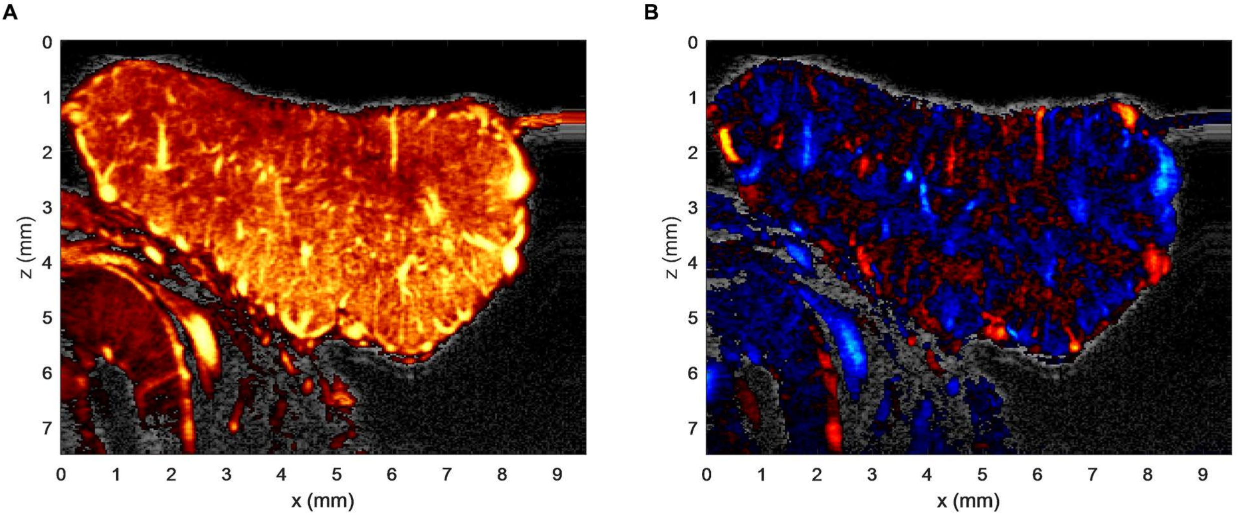
A
B
z (mm)
x (mm)
0
1
2
3
4
5
6
7
8
9

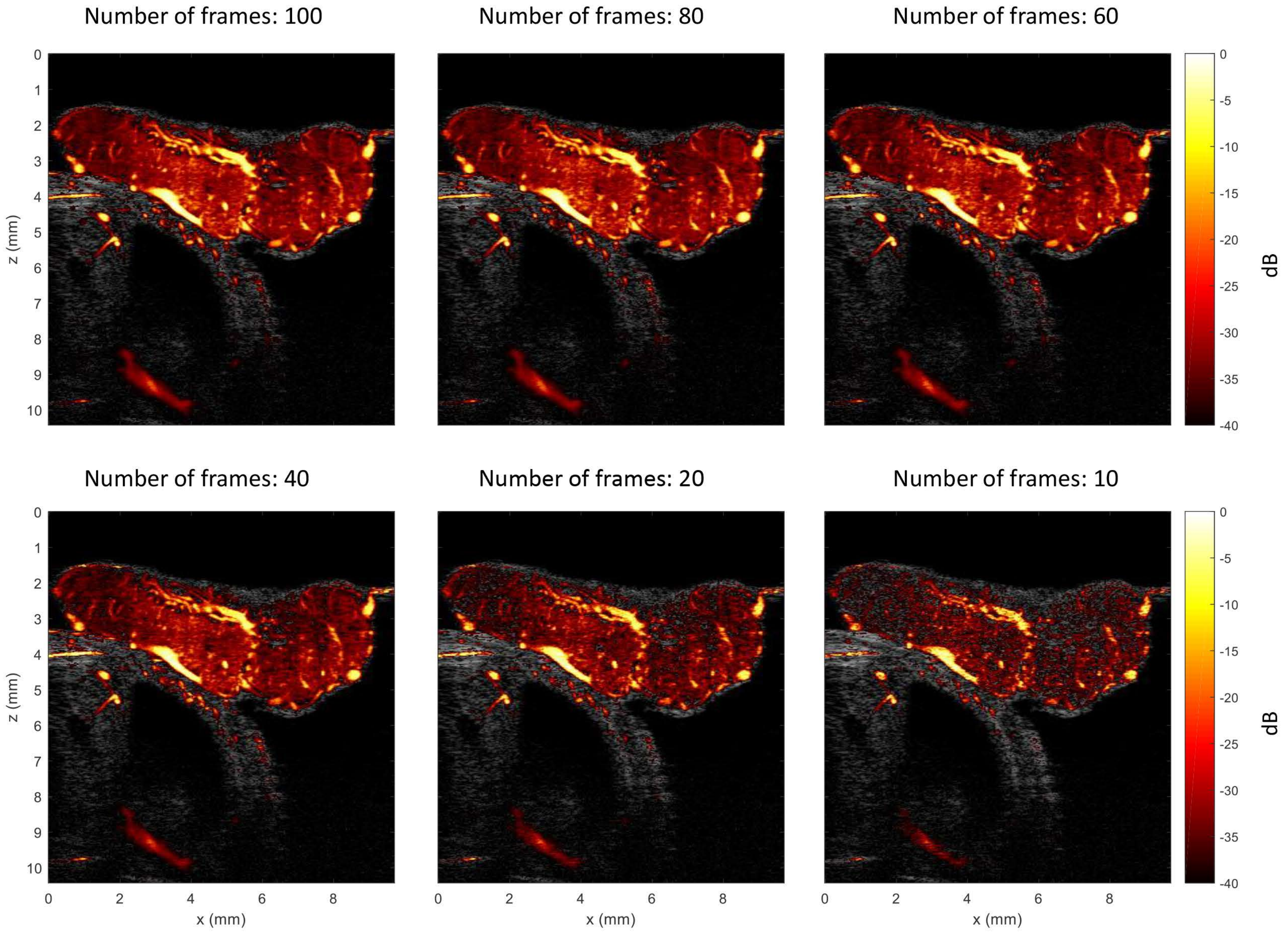

Number of frames: 100
Number of frames: 80
Number of frames: 60
Number of frames: 40
Number of frames: 20
Number of frames: 10
z (mm)
x (mm)
dB
0
-5
-10
-15
-20
-25
-30
-35
-40
